# Supplementary material for: Inhibition of Hepatic AMPK Pathway Contributes to Free Fatty Acids-Induced Fatty Liver Disease in Laying Hen
Source: Metabolites. 2022 Sep 1;12(9):825. doi: 10.3390/metabo12090825 (PMC9502618; doi:10.3390/metabo12090825)
Supplement: Supplementary file 1 [file metabolites-12-00825-s001.zip › metabolites-1830433-supplementary.pdf]

Inhibition of Hepatic AMPK Pathway Contributes to Free Fatty Acids-Induced Fatty Liver Disease in Laying Hen

Cheng Huang [1,†], Xiaona Gao [1,†], Yan Shi [2], Lianying Guo [1], Changming Zhou [1], Ning Li [1], Wei Chen [1], Fan Yang [1], Guyue Li [1], Yu Zhuang [1], Ping Liu [1], Guoliang Hu [1] and Xiaoquan Guo [1,*]

[1] Jiangxi Provincial Key Laboratory for Animal Health, College of Animal Science and Technology, Jiangxi Agricultural University, Nanchang 330045, China
[2] School of Computer and Information Engineering, Jiangxi Agricultural University, Nanchang 330045, China
* Correspondence: xqguo20720@jxau.edu.cn; Tel.: +86-791-8381-3345
† These authors contributed equally to this work.

The following Supporting material is intended for publication

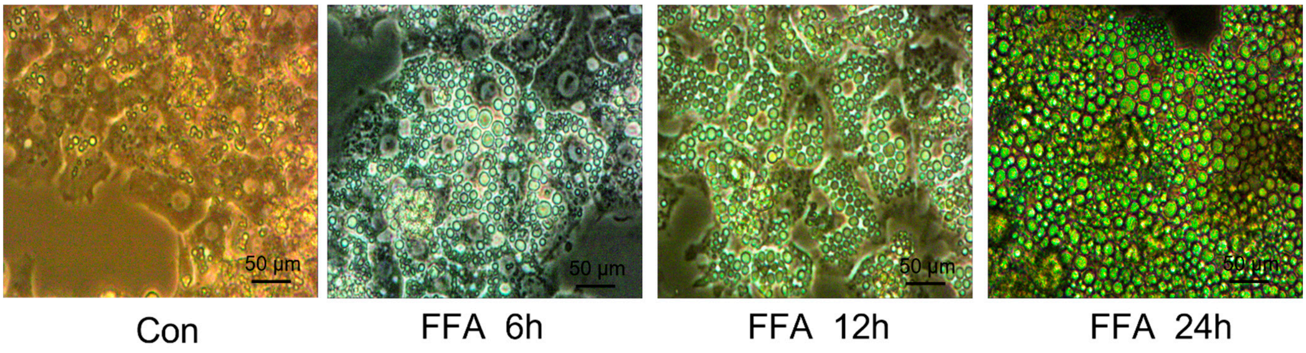

Figure S1. FFA-induced fat accumulation in chicken hepatocytes. The morphological changes of the cells were observed under an inverted microscope in the normal group, FFA induced 6h, 12h, and 24h in the FFA group.

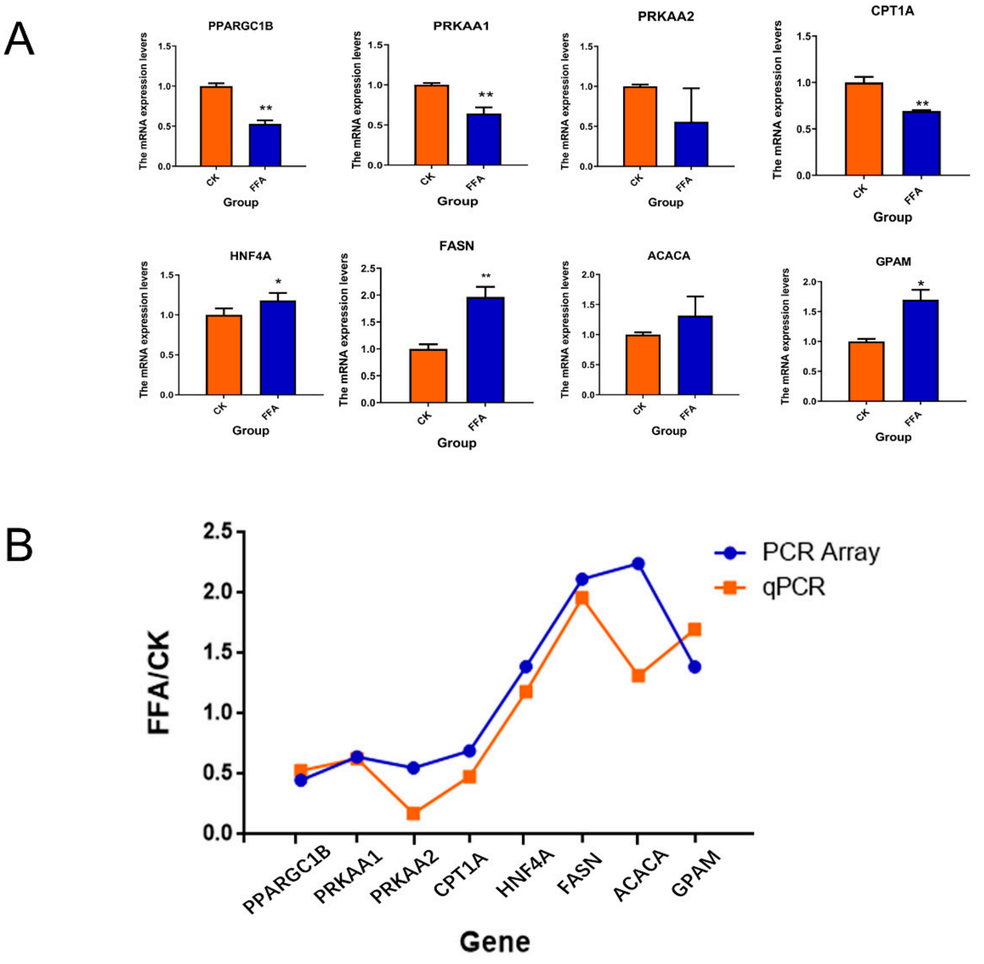

Figure S2. Effects of FFA treatment for 24 h on the transcript levels of genes related to primary hepatocytes in laying hens. (A) Gene expression levels of PPARGC1B, PRKAA1, PRKAA2, CPT1A, HNF4A, FASN, ACACA and GPAM, respectively. (B) The fitting curve of RT-PCR and validation of PCR Array. Data are shown as mean ± SD (n = 6), where (*) $P < 0.05$ and (**) $P < 0.01$.

Table S1. The sequence of target genes primer.

Gene names	Sequence of primer (5'-3')
ADRA1A	F:GTCATGCCTCTTGGTTCTTTCT
	R:GCTGGGATGGTTGAGGTTGA
ADRA1B	F:CCACTAGGATCTCTGTTTTCAGC
	R:AGCACCGATTCCAGTTCCTG
ADRA1D	F:TCGTCCTGCCTTTTGGTTCTTT
ADRA2A	R:AGTAGCCTGATGAACGCTCG
	F:TTGCTCATGCTCTTCACCGT
ADRA2B	R:ACATCCAGGGCCAGGTAGAT
	F:CCCGAGAAATCAGGGGCAAT
ADRA2C	R:TTGTTAAAAGGCGGCAGTCG
ADIPOR1	F:TCTTCAACCAGGACTTCCGC
	R:CTATGCCCCAACCCCATGTT
ADIPOR2	F:GCTGGTTCTTCCTCATGGCT
	R:TGTGACTGGAACCAGATGTCG
INSR	F:ATAGGACCGGGAGGCGGTA
LEPR	R:GATTATGCGTTGGCTCTGGC
	F:ACCCGACGCTGAGAATAACC
CHRNA1	R:AGCACCTGTTCGTTGGAGAG
AK1	F:AATGACTGGCAGGTGGTTCA
	R:TTCTTGGACGCTACGCTCTG
AK2	F:TGTGCTGCTCCTGATCTTCTCC
	R:GTTTATGTCTGTCCATTGCTGCT
AK3	F:CAGCAGCATGTCGACAGAAAA
AKT1	R:GCCCTTCTCCATGATGGCTT
	F:ATGCCTCCCAGTCTCCTGAT
AKT2	R:CGGAGTCACTCAGATGTTGCT
	F:ACATCATGACACGGCTGATACT
AKT3	R:CATCAATGCCCTGCACTTTGG
PDPK1	F:CCTTTTGTGGACCCTTCTGC
	R:AGAAAATACCGTGGCCTCCA
CAMKK1	F:CGGGCACATTAAGATCACCG
	R:TCCTGGTTGTAGAAGGGCAG
CAMKK2	F:TGGTCCGAGAGAAGGCTAGT
PRKAA1	R:ATCTTCCAACACCTCTCCTCCAT
	F:CTGGGCTCTTAAAGGGCGAC

PRKAA2	R:AGCATCATACAGGTGGCTGC
	F:CTGAGCGCAGATATTTTGAGG
PRKAB1	R:CAAAACCCGGAGTGTTGCTC
	F:ACGCTCTCAGAAACCAGGAA
PRKAB2	R:TAACTTCTGGCTGGTCTGGG
PRKAG1	F:TCCGAGACATTAGGGAACACG
	R:GCGAGACAGATGGTGATCGT
PRKAG2	F:GGCAAAGTCAAGGTTGGCGA
	R:GAAGAAGTCTGTTGGCGTGC
PRKAG3	F:CTGAGAGCCGTCGCTTCTT
PRKACA	R:TATCCAATGGCGCCTTCATCT
	F:AGAAACCTCAGGTCGGGACT
PRKACB	R:GCCGGGTCACACGAGATATT
	F:TTCCGGTCACGTGTGGAGTA
PRKAR1A	R:TGGTCAGCATGCCCACAAAG
PRKAR1B	F:TGGATGCTCATCACGTCTCC
	R:AGGCCTTCTTTACTTGTAGCGT
PRKAR2A	F:GACATCATCGACCGCATCAC
	R:AGGGTGCGAATGGAGAAAGA
PRKAR2B	F:TCGGGATGGGATGAGGAGTT
PPP2CA	R:TTTGTACCCCCTGTCTCCCG
	F:GAGATCGAGAGCGGCATTCA
PPP2CB	R:AGGAACTCTTTCACAAAGGTCT
	F:ATCTCTGGACAAGTGGGAGC
HMGCS1	R:CGTTTTCTGATCGACGCTGT
PPP2R2B	F:CGTCGCGTGCTTTAATGCTT
	R:ACAGTAAACAGACCGTTGCAGA
PPP2R4	F:CATGAGCATCGAGATTCCGC
	R:CGGTTAATGACTGGAGGCTCG
ATG13	F:TCCGAGCGTTTGAAAGTGGT
RB1CC1	R:TGCACGTCCATAACTTCCTTGT
	F:CCTTGGTGGATGGCCAGATTT
ULK1	R:GCAAGCGATCAAGTGCTCTG
	F:TACGTTGCCCTGTCACTGTC
ACACA	R:GGGTAACGCACCTTTAACGC
ACACB	F:GTTGCTATGCTAGTTGGGTCAA
	R:CCACCGACTTCTGTACCAGT
CPT1A	F:GACAGACGGGCGATTCACA
	R:CAGCAATTCTCCGGTGTGGT

CPT2	F:TGGAAGCGCTCTCAGGCATA
FASN	R:GGCTTTGTTCCCAAAGCGAG
	F:TAGGTGGACACCAGACTGCC
GPAM	R:GTCGGGCCTGGACAATTACT
	F:AGCGGCTGACGACAATAACA
GPAT2	R:ACCAGAGAAGATCCGAGGGT
HMGCR	F:CAGCCCATCCCCAGTGATTT
	R:GCTGGAAGACCCCACTGAAA
LIPE	F:CCGGCACCCTGAGTTCATT
	R:GCAGACAGAAACAGGGGACA
MLYCD	F:CCGTGAGGAACCAATCCACA
PNPLA2	R:GGAATTCTCTCTGCTGGGCA
	F:GAGCTGTTCAGAACTCTCTCCA
GYS2	R:AGCCTCATGACTCATTCGCA
	F:GATACGTGGGCATTGCTGAG
PFKFB2	R:CGCCCCAGGAATGTTCAAAA
PFKFB3	F:GCTAAGATGGCATTGCACGG
	R:TGCCAGAGCCTCCACTATCT
PFKFB4	F:GCCCTGTCGTGGGAAATCA
	R:GATCAGATGCTTAAGTGCTGAATCT
SLC2A4	F:TTCCTCTCCACCCACAAGTC
CAB39	R:CAATGCCTGTTCCACTCTCG
	F:GCCGTATTCTTGCAGATGGG
MTOR	R:CATTCCCATTGCATCCCCTG
	F:CCATCGGGTTGGTGTCCTAC
RPTOR	R:CTCTTCCAGAAGCCCACGTC
STK11	F:AGGGAGTGGAACTTGGGACT
	R:TCTGTGAGGAGAGGAGACCG
STRADA	F:TGGACTCCGCTTGGAACATC
	R:CCAGGAACCTCTTTCGTGCT
STRADB	F:GGAGTTTGCTCAGGGCAGTG
TSC1	R:CCCACTGAGGAAGCCCATTA
	F:TGAACTCAAGGCTGGCTGG
TSC2	R:AGCAGCTGAAAAATCTTCGCA
EEF2K	F:AGCGTCCAATCTTGATGCCA
	R:TTGGGACACCGATCCAGTTG
EIF4EBP1	F:GGGACAGCTGACTCAGAACC
	R:CTGCATGGCCTCCTCGTTAT
RPS6KB1	F:GGTTGACAGGGAACCCTACG

RPS6KB2	R:CGCTCCCCTCGTAAAGTTGT
	F:TGACTCTTCCTGGACGTTGC
CRTC2	R:AAGCCTTACGGTAACCTGGC
	F:ACTCTGCTAGCAAACGACCC
CRY1	R:CACAGCAGTTTGGCAAGGTC
ELAVL1	F:TAGAAGACTGCAAGCCCAGC
	R:CACATCAGGAGGGTCCACAC
FOXO3	F:TGGTGCCCATACCTCCTAGC
	R:TCCGTCCCGTTCATGCAGAT
HNF4A	F:CTTTTCTTCGGTGGGTGTCTGAA
PPARGC1A	R:GAGCTCGCCTCATTTGTCTC
	F:CGGCGACGGGTGTAACAA
PPARGC1B	R:AAGAAGAAGTCCGTGGCAGG
	F:TGACTGGACTCACTTTGGAGATG
SREBF1	R:AGCCACTATCCCTTCGTGCT
TP53	F:GGACAGGGTGAGAGATTAGGG
	R:TGTGGACCATGTCAGCTACG
ACTB	F:TCCCGCCTGTCTGAAAACTC
	R:CTCCACACTTATCTTCTCTCTATGA
GAPDH	F:CGGGCGGAACCAGGATTATT
PPP1R12A	R:GTCCGGAAGGTCAGAAGGTG
	F:TACTTAGCGCAGCGAGGAAG
TXNIP	R:CTCCAGCTCCTCATCCGAAC
	F:ATCCCTTCTTCCGGCACATC
LOC107057170	R:GTGTGGGGGCTGCTGTT
CRTC1	F:CAACGTCAATCAGATCGGCA
	R:CGAAGGCAAACGGAACAGATG
	F:ATGGTCAGACAGTTGGCGTT
	R:TTCGGCCCAACACTTTGAGT
	F:GTTTGACAAAAGGTCAGAAGCAGAA
	R:CACCCATAGGAGAGAACCTGA
	F:AGAGCTATCTGCAGGTGGGA
	R:ACGCGAGTGATTTCACGACT
	F:CTAAGCGCTAAGATGGAGGCT
	R:CCTTCTTCATCCCTGCTCGG
	F:CATGTGCAACCAGGACTCTGT
	R:AGGCTCATTGCTGTACTGGC
	F:AGGCAGTATGACCAAAGCCC
	R:AGCAGTGAGAAACGAGTGGG

	F:AAGGGGTCTGACACATGGAG
	R:GGGGAGGTCTTGTGAATGGA
	F:AACCATTGCTGGAACCCACT
	R:GCCAGTTGCTGTGATCCTCA
	F:CACAGATCATGTTTGAGACCTT
	R:CATCACAATACCAGTGGTACG
	F:GGCACGCCATCACTATC
	R:CCTGCATCTGCCCATTT
	F:GGATCACTCAGTGTGTCTTCG
	R:GTAACTGTGCTTTCAGCTTTTCA
	F:GTGCGTAAACTGAAGCCGTC
	R:TCGGGGACGATGTCCAGATA
	F:TGGGGTTGGACACACGTAAG
	R:GTGGTCCACGATGGTCACTT
	F:GCCGTATTTTGCCCCTTGTC
	R:GCACCCCCTTCTAGTGCAAA
